# Miscellaneous Intelligence, Observations, and Hints

**Published:** 1800-12

**Authors:** J. C. Lettsom

**Affiliations:** London


### MISCELLANEOUS INTELLIGENCE, OBSERVATIONS, AND HINTS.

To Dr. BRADLEY.

Dear Sir,

Having been politely favoured with a view of the accurate ftatement fent to your valuable Publication, refpecting fome fuppofed cafes of Cow-pox, which happened in the neighbourhood of town, I beg to inform you that I had previoufly feen thefe unfortunate fubjects, and fince repeated my vifit; and from infpection, and the whole hiftory of the cafes, am of opinion with thofe refpectable gentlemen who figned the ftatement, that the difeafe was not the cow-pock, but morbid ulceration, originating from the purulent matter formed under the fcab, or dried puftule of the cow-pock, with which the patients were infected.

Before I had feen the ftatement, I had made enquiries refpecting the origin of the matter from whence thefe were inoculated, and have no doubt of its having been the genuine cow-pock with which the child of my next door neighbour was inoculated; and from this my own grand-child, and the child of a refpectable furgeon of my acquaintance, were inoculated, as well as the children of feveral other families, not one of whom fuffered under any complaint requiring medical affiftance; and yet from one of thefe was this purulent matter unfortunately taken. I have therefore no reafon, from any thing connected with the cow-pock that ever came under my obfervation, to change the high opinion I entertain in favour of this new mode of obviating one of the moft fatal difeafes to which mankind are liable.

I am, refpectfully, &c.

*London, Nov.* 25, 1800. J. C. LETTSOM.

---

*The two following Extracts are taken from Letters addreffed to Mr. Chamberlain, Surgeon, of this City, viz.*

Sir, *Delvine, Aug.* 24, 1800.

As it is two years fince I applied to you for fetæ of cowhage, you muft have been led to imagine, that I did not find it ufeful to my poor patients, that I never wifhed for more; but the truth is, that I approved of it fo much, that I employed a druggift at

 Edinburgh

Edinburgh to provide it for me, and have made ufe of a very confiderable quantity. I find the account you give of its innocence is perfectly juft, and that where it does no good it never does any harm. It does great fervice in fevers, even where infection and not worms is the primary caufe of the difeafe; and amongft the commonality, who live moftly on vegetables, fevers and coughs are very frequent during lying-in, and I have four or five times given your electuary in fuch cafes, and always with the beft effects. In this country, delirium or derangement of the melancholy kind is a common difeafe, and I find that worm medicines never fail to do good; my ufual prefcription in a very bad cafe, is firft, tartar emetic, then cowhage in much larger dofes than what you recommend."

*Kingston, Jamaica, Sept.* 15, 1800.

I am aftonifhed to hear that fome phyficians in England have written to their friends here of the "madnefs of our practice." I fhall not call them mad; but I think them blameably prefumptive in writing of what they know nothing at all. For, if they are eminent, their opinions may poifon the minds of fuch unfortunate ftrangers as may be taken ill, fo as to make them object to the only treatment that has a chance of faving them from the moft fatal of all difeafes yet known, fcarcely the plague itfelf excepted. I will aver that a Cullen, or any other high medical character, never having feen the difeafe, would be puzzled and baffled if he purfued any other courfe. Mercury, we know, can cure many other difeafes without its peculiar action on the falivary glands; but here it will not: for I have not yet known an inftance but one which has been cured without falivation; and never have feen or heard of one that died, when that difcharge could be produced. In this folitary cafe the mercury (fay 700 or 800 grains of cal. befides feveral ounces of merc. oint. by frictions) feemed to act by profufe perfpiration, as it had no other effect whatever. I have reafon to think that it operates fo in the cures; becaufe, when it was counteracted by bark, which was infifted on by a confulting phyfician, the patient had a return of bad fymptoms, which were banifhed by a difcontinuance of the bark, and a perfeverance in the ufe of mercury, until the patient got well. Bark, though an excellent medicine in proper cafes, has fent many to the grave, whom, in all probability, mercury would have faved. Therefore, my friend, whenever you may have an opportunity of giving advice to adventurers coming this way, never neglect to recommend having the falivary glands flightly affected by mercury before they embark, or immediately on their arrival on the fpot of infection. I have had fo many inftances of the good effects of prophylaxis, that I can recommend it with confidence. Indeed it is a duty which I, and I truft you, think indifpenfable to perform for the good of our fellow creatures. It is a lamentable fact to acknowledge, that notwithftanding this only yet known remedy for this dreadful fcourge of new comers, it fails too often after the commencement of the difeafe; although it has done wonders in very defperate cafes, after late application.

To

To Dr. BRADLEY.

DEAR SIR,

At the conclusion of your last Number I observe, that Dr. Nisbet has introduced my name as a collateral support to his narrative of a case of cancer recently cured. I *once* visited the patient alluded to, and have no objection to give you MY OWN statement of what I then saw; but as I know nothing of the progressive stages of the complaint, nor of the medical treatment, which remains a profound secret, my testimony can serve no good purpose.

I remain, &c.

*Great Russell Street, Bloomsbury,* WILLIAM BLAIR.

*November*, 3, 1800.

---

*A Sketch of the University of Cambridge, Massachusetts, founded 1630, with some Account of its Medical School.*

I have observed in your very valuable volumes of the Medical and Physical Journal, mention made of several American medical schools, lectures, &c. while the one at Cambridge, the most celebrated in the western world, passes without much notice, although it has flourished more than a century before most of those you mentioned had an existence. Cambridge is the mother seminary of the United States, and was founded soon after the settlement of the country, by persons educated at your Oxford, and particularly your *Cambridge*; its professors came directly from these universities in the reigns of Elizabeth and James. The university library contains about 14,000 volumes, and its philosophical apparatus is by far the largest in the United States. You may see a particular account of it in the last edition of Dr. Morse's Geography.

The medical lectures, or rather the medical school, ingrafted on this university, is of no longer date than 1783, although this university, originally called *Havard College*, was founded in 1630. Medical students come from distant states, and even from Canada, to attend the medical lectures in this place. —— Nichols, Esq. member of parliament, son of Dr. Frank Nichols, and grandson of Dr. Mead, has just presented our medical school with his father's collection of anatomical preparations. Some curious representations in wax have also arrived from Italy, so that the medical branch is in a very flourishing condition.

The university of Cambridge is three miles from Boston, and is remarkable for the beauty and healthfulness of its situation: It is intimately connected with the government of the commonwealth, for the governor, the council and senate of the commonwealth, together with the clergy of Boston, and the six neighbouring towns, are, ex officio, the governors of it. The six other colleges in New England have grown out of this as branches from a trunk.

*Sept.* 1800. AN AMERICAN.

---

Any

Any thing which has a tendency to cure or even to relieve perſons afflicted with Epilepſy, is entitled to attention; the following lines on the ſubject are quoted from LALANDE. "There was lately brought to Citizen PORTAL, a young lady who was every day attacked by violent epileptic fits. They began in one of her toes; which circumſtance ſuggeſted to that able anatomiſt the idea of cutting the nerve, for the purpoſe of interrupting the communication: but he began by the application of *opium* to the nerve; and that alone proved ſufficient to effect a complete cure."

---

A very curious and ſcientific paper has appeared in the laſt volume of the Philoſophical Tranſactions of the Royal Society of London, by Mr. ASTLEY COOPER, ſurgeon, on the effect produced on the ſenſe of hearing, by a perforation or entire loſs of the *membranæ tympani* (*drum* of the ear). It has generally been imagined that ſuch an accident would be attended with complete deafneſs, but ſeveral caſes here related, ſhew that the loſs of this ſenſe is but very partial, and ſometimes even ſo little as to produce but ſlight inconvenience. A perforation of this membrane is indicated when air or ſmoke can be driven from the mouth through the external ear.

---

Dr. GUTHRIE, of St. Peterſburg, in a letter to Profeſſor DUNCAN, of Edinburgh, mentions a curious remedy, which has performed the cure of a dropſical caſe, which was, to ſwallow daily a table-ſpoonful of common ſand. This remedy was found to purge pretty briſkly, which was followed by a relief of all the ſymptoms.

---

### VACCINE INOCULATION.

We obſerve that in ſome large cities the Faculty have been induced to ſign a public teſtimonial of their approbation of the Vaccine Inoculation. The names of theſe Gentlemen in the Town of LEEDS, and Cities of DURHAM and CHESTER, have come to our hands, and we have great pleaſure in ſubjoining them.

IN LEEDS BY

Stanhope Baynes, M. D.

Robert Daviſon, M. D.

Benjamin Hird, M. D.

R. W. D. Thorp, M. D.

Joſhua Walker, M. D.

Obadia Brooke, Surgeon.

Thomas Chorley, Surgeon.

T. A. Coates, Surgeon.

Sam. Dickenſon, Surgeon

Wm. Dodſworth, Surgeon

William Hey, Surgeon

William Hey, jun. Surgeon

Maurice Logan, Surgeon

John Moxon, Surgeon

Benj. Muſgrave, Surgeon

Tho. Parkinſon, Surgeon

John Robinſon, Surgeon

Thomas Ruſby, Surgeon

Matthew Shirtliffe, Surgeon

John Soper, Surgeon

James Tatham, Surgeon

Thomas Teale, Surgeon

IN DURHAM BY

G. Cayley, M. D.
Potts and Clifton, Surgeons
J. James, Surgeon
W. Green, Surgeon
W. Ward, Surgeon
G. Fothergill, Surgeon
W. Ruddock, Surgeon
—— Nelſon, Surgeon

In

IN CHESTER BY

| | |
|---|---|
| William Currie, M. D. | P. Wilkinfon, Surgeon |
| William Houghton, M. D. | C. Tomlinfon, Surgeon |
| W. M. Thackeray, M. D. | Geo. N. Hill, Surgeon |
| Jas. Arden, M. D. | John Harrifon, Surgeon |
| D. Orred, Surgeon | J. Okell, Surgeon |
| G. Rowlands, Surgeon | W. Connah, Surgeon |
| S. Freeman, Surgeon | Chrift. Buck, Surgeon |
| Geo. Harrifon, Surgeon | J. M. Afsheton, Surgeon |

Meetings of the Faculty have alfo been held at *York*, *Hull*, *Birmingham*, and fome other places, and a fimilar refolution adopted.

---

Notice refpecting a new Pharmacopœia, by Mr. Reece, Fellow of the Royal College of Surgeons in London, and late domeftic Surgeon and Apothecary to the General Infirmary at Hereford.

To the EDITORS of the MEDICAL and PHYSICAL JOURNAL.

Gentlemen,

THE prefent melancholy declining ftate of almoft every charitable public inftitution for the relief of the difeafed poor in this kingdom, through the unparalleled preffure of the times, has induced me to offer to the phyficians and furgeons of thofe inftitutions, a collection of cheap officinal and extemporaneous Recipes, entitled, "The MEDICAL and CHIRURGICAL PHARMACOPOEIA for the Ufe of HOSPITALS, &c." which, through the medium of your very valuable Publication, I beg leave to recommend to the notice of the medical tribe. Thefe medicines, if adopted, will reduce the expence of the Difpenfary in the immenfe proportion of eighty per cent. and experience warrants me in afferting them to be equal in efficacy to thofe of the moft expenfive clafs.

Although I have more particularly publifhed this work for the ufe of hofpitals, &c. I flatter myfelf it will be found of equal utility in private practice, more efpecially to thofe gentlemen who exercife, at very inadequate falaries, the offices of Surgeons and Apothecaries to parifhes; the prefent expenfive formulæ preventing their doing juftice to the unfortunate objects labouring under the complicated evils of poverty and difeafe.

It was my intention to have annexed cafes of the fuccefs of each particular medicine, which I relinquifh from the great augmentation it would be to the fize and confequent price of the book; but I beg to affure the Faculty, that they have feverally undergone the teft of experience with the moft flattering effect.

I am, &c. &c.

*London, Nov.* 13, 1800. RICHARD REECE.

---

Mr. STEPHEN HAMMICK, Member of the Royal College of Surgeons, London, and Affiftant Surgeon to the Royal Hofpital, Plymouth, propofes, on Monday the 5th of January, commencing a Courfe of Lectures on the Principles, Practice and Operations of Surgery. Particulars known at his houfe, Parade, Plymouth.

Dr. Harrington has in the prefs, and will fpeedily publifh, fome Experiments and Obfervations on Volta's Galvanic Pile, clearly elucidating all the phenomena.

---

The *Medical and Phyfical Journal* continues to be tranflated into German, and re-publifhed at Leipfic, by the bookfeller Sommer. We are informed the Tranflator is Profeffor Kühn of Leipfic.

